# Machine learning reveals distinct gene expression signatures across tissue states in stony coral tissue loss disease

**DOI:** 10.1098/rsos.241993

**Published:** 2025-07-23

**Authors:** Kelsey M. Beavers, Daniela Gutierrez-Andrade, Emily W. Van Buren, Madison A. Emery, Marilyn E. Brandt, Amy Apprill, Laura D. Mydlarz

**Affiliations:** ^1^ The University of Texas at Austin Texas Advanced Computing Center, Austin, TX, USA; ^2^ Department of Biology, The University of Texas at Arlington, Arlington, TX, USA; ^3^ Department of Integrative Biology, Michigan State University, East Lansing, MI, USA; ^4^ Center for Marine and Environmental Studies, University of the Virgin Islands, St Thomas, VI, USA; ^5^ Marine Chemistry and Geochemistry Department, Woods Hole Oceanographic Institution, Woods Hole, MA, USA

**Keywords:** stony coral tissue loss disease, coral, gene expression, machine learning, transcriptomics, symbiosis

## Abstract

Stony coral tissue loss disease (SCTLD) has rapidly degraded Caribbean reefs, compounding climate-related stressors and threatening ecosystem stability. Effective intervention requires understanding the mechanisms driving disease progression and resistance. Here, we apply a supervised machine learning approach—support vector machine recursive feature elimination—combined with differential gene expression analysis to describe SCTLD in the reef-building coral *Montastraea cavernosa* and its dominant algal endosymbiont, *Cladocopium goreaui*. We analyse three tissue types: apparently healthy tissue on apparently healthy colonies, apparently healthy tissue on SCTLD-affected colonies and lesion tissue on SCTLD-affected colonies. This approach identifies genes with high classification accuracy and reveals processes associated with SCTLD resistance, such as immune regulation and lipid biosynthesis, as well as processes involved in disease progression, such as inflammation, cytoskeletal disruption and symbiosis breakdown. Our findings support evidence that SCTLD induces dysbiosis between the coral host and Symbiodiniaceae and describe the metabolic and immune shifts that occur as the holobiont transitions from healthy to diseased. This supervised machine learning methodology offers a novel approach to accurately assess the health states of endangered coral species, with potential applications in guiding targeted restoration efforts and informing early disease intervention strategies.

## 1. Introduction

Coral reefs are among the most threatened ecosystems on the planet. Anthropogenic ocean warming, among other stressors, has triggered mass bleaching and disease outbreaks, resulting in substantial coral cover and reef biodiversity loss on nearly all the world’s tropical coral reefs [1–6]. Recovery windows between stress events have also narrowed at an unprecedented rate [7]. This limits the ability of reefs to recover without intervention, highlighting the importance of coral reef management efforts as well as meaningful action on climate change [8–10]. Compounding these global stressors, emerging coral diseases have intensified reef decline, posing new challenges for conservation and management. Recent efforts have focused on the identification of coral species, individuals, symbiont genera, genetic mutations and gene expression patterns that can withstand multiple types of stressors [11–16]. The rapid expansion of high-throughput (omics) datasets, such as genomics, transcriptomics and metabolomics, and the advancement of novel statistical methods, such as machine learning (ML), hold promise for the accurate identification and characterization of emerging and persistent threats to coral reef ecosystems.

One disease event that has significantly altered coral reef assemblages and functionality is stony coral tissue loss disease (SCTLD) [17–21]. First observed off the coast of Miami, Florida in 2014, SCTLD has led to significant losses of coral throughout Florida’s coral reef and the wider Caribbean [22–27] and is the most pervasive and virulent coral disease on record. Despite research efforts, the aetiology of SCTLD remains unknown, probably due to the complexity of microbial and eukaryotic assemblages that associate with coral [1,28–30]. However, shifts in the bacterial [31–38] and viral ([39,40], but see [41]) consortium have been implicated in SCTLD progression, and antibiotic treatment has proven effective at halting active lesion progression [42–45]. Furthermore, histopathology has identified SCTLD tissue necrosis originating in the coral’s gastrodermis where their algal endosymbionts (Symbiodiniaceae) reside [46]. Evidence of symbiont necrosis and other morphological abnormalities consistent with Symbiodiniaceae pathology has also been observed in both apparently healthy and SCTLD-affected corals [39,46]. Together, these results support the possibility that Symbiodiniaceae play a central role in SCTLD disease progression.

Recent omics analyses have provided further insight into the cellular mechanisms underpinning SCTLD pathogenesis. Metabolomics on apparently healthy and SCTLD-affected corals has revealed variations in Symbiodiniaceae-derived lipid and tocopherol production in response to disease [47], supporting the case for Symbiodiniaceae involvement. Transcriptomic analyses have identified signatures of *in situ* degradation of photosynthetically dysfunctional Symbiodiniaceae [48] as well as commonly differentially expressed genes (DEGs) involved in innate immunity, apoptosis and extracellular matrix (ECM) structure in SCTLD-affected corals [49,50]. Interestingly, paired *ex situ* transmission and *in situ* intervention experiments showed that amoxicillin treatment led to a ‘reversal’ of many of these signalling pathways, suggesting that disease intervention provides benefits to the coral beyond the removal of pathogens and opportunistic microbes [50]. Despite these advances in our understanding of SCTLD, there is still a need to identify the central processes involved in SCTLD progression to assist coral preservation and restoration projects.

Next-generation sequencing technologies have revolutionized disease research, allowing scientists to measure expression-level changes of thousands of genes in response to experimental or natural infection [51]. However, gene expression datasets are high-dimensional, and the DEGs produced often contain redundant and biologically irrelevant data [52]. Integrating supervised ML, defined as the process of learning from labelled examples to predict or classify an outcome of interest [53], into DEG analyses offers a promising solution to address the ‘curse of dimensionality’ [54] prevalent in large omics datasets. One widely adopted supervised ML algorithm for feature selection is support vector machine recursive feature elimination (SVM-RFE). SVM-RFE reduces data complexity by identifying and ranking genes based on how well they distinguish between phenotypes (e.g. tissue states) and has many applications in gene expression research. For example, SVM-RFE has been used to predict drought-resistant genes in *Arabidopsis thaliana* [55], identify genes for accurate cancer classification [56] and diagnose Alzheimer’s disease in humans [57]. Broadly, feature selection using SVM-RFE has been shown to be successful at isolating a subset of non-redundant and biologically relevant genes from a larger dataset, enabling the construction of robust models capable of accurately assigning data points to predefined classes.

Here, we utilize a supervised ML approach aimed at characterizing the gene expression associated with various tissue health states in a major reef-building coral, *Montastraea cavernosa,* and its dominant algal endosymbiont, *Cladocopium goreaui*. Through the use of the ‘sigFeature’ feature selection algorithm [52], we implement a simultaneous combination of SVM-RFE and differential expression (DE) analysis to identify genes that are both biologically relevant and have the highest discriminatory power within three types of coral tissue collected from a natural reef environment: apparently healthy tissue on an apparently healthy colony (HH), apparently healthy tissue on an SCTLD-affected colony (HD) and lesion tissue on an SCTLD-affected colony (LD). This approach allows us to pinpoint the most relevant genes associated with different states of SCTLD progression, thereby enhancing our understanding of its pathogenesis and supporting the development of effective treatment and prevention strategies.

## 2. Methods

### 2.1. Sample collection and site description

Coral fragments approximately 5 cm in diameter were collected by divers on SCUBA with hammers and chisels from two reefs in St Thomas, United States Virgin Islands (USVI) showing signs of active SCTLD in February of 2020: Buck Island (18.27883°, −64.89833°) and Black Point (18.3445°, −64.98595°). Black Point, a nearshore reef, first exhibited SCTLD cases between December 2018 and January 2019 [27]. Buck Island, situated near an offshore, undeveloped island, recorded its first cases of SCTLD in October 2019. Current environments at both sites are similar. SCTLD-affected corals were identified based on displaying acute multifocal lesions consistent with the SCTLD case definition [23]. Lesions were bright white where the skeleton had recently been denuded of tissue, with no visible algal colonization at the skeletal/tissue boundary, indicating actively expanding lesions.

**Figure 1.**
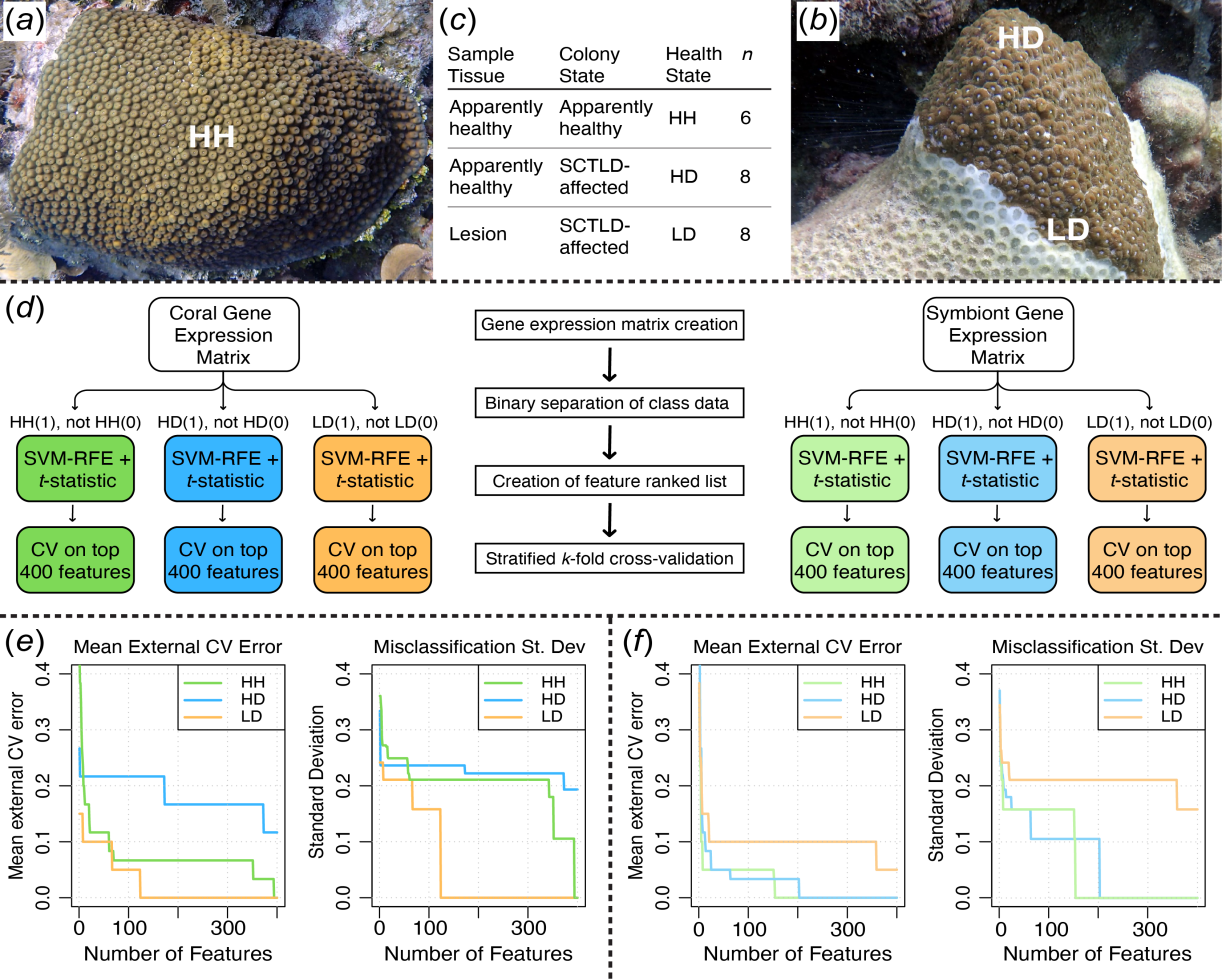
Experimental design and analysis workflow. (*a,b*) Photographs showing the three tissue health states collected: HH, HD and LD. (*c*) Sampling data. (*d*) Feature selection pipeline using a combination of SVM-RFE and DE *t-*statistic with the sigFeature R package [52]. (*e*) External stratified *k*-fold cross-validation (CV) results on the top 400 *M. cavernosa* features from each tissue health state. (*f*) External stratified *k*-fold CV results on the top 400 *C. goreaui* features from each tissue health state (SVM-RFE, support vector machine recursive feature elimination; DE, differential expression). (*a,b*) Photo credit: Amy Apprill.

At both sites, one coral fragment was collected from each apparently healthy colony (Buck Island, *n = 3*; Black Point, *n* = 3), termed HH. Two fragments were collected from each diseased colony: one immediately adjacent to the SCTLD lesion line (Buck Island, *n* = 3; Black Point, *n* = 5), termed lesion tissue on an SCTLD-affected colony (LD), and one as far away from the lesion line as possible (approximately 10 cm from the lesion line; Buck Island, *n* = 3; Black Point, *n* = 5), termed apparently healthy tissue on an SCTLD-affected colony (HD). The sampling scheme (shown in figure 1*a*–*c*) aimed to capture the variability in gene expression across different tissue health states while ensuring consistency in sampling methodology. Coral fragments were placed in individual bags that were sealed and transported to land on ice before being flash-frozen at −80 °C.

### 2.2. RNA extraction and sequencing

Total RNA was extracted from all 22 coral fragments following a protocol outlined previously [48] using the RNAqueous-4PCR Total RNA Isolation Kit from Invitrogen (Life Technologies AM1914). About 1 g of frozen coral tissue was scraped off each fragment into a 2 ml microcentrifuge tube using a sterilized bone cutter. For LD samples, tissue was deliberately collected from visibly pigmented tissue adjacent to the actively expanding lesion boundary. Areas that appeared fully bleached or visibly degraded were excluded to avoid confounding effects related to tissue necrosis or symbiont loss. Lysis buffer was added to each microcentrifuge tube followed by mechanical disruption using a refrigerated Qiagen Tissuelyser II at 30 oscillations per second for 60 s. Elution was performed in two 30 µl steps at a time. After combining elutions, contaminating DNA and chromatin were removed using the Ambion DNase I kit from Invitrogen (Life Technologies AM 2222). Resulting total RNA samples were sent to Novogene Co., Ltd. (Beijing, China) for quality assessment using an Agilent Bioanalyzer 2100. All samples passed quality assessment with RNA integrity values ≥ 7 and were preprocessed for mRNA enrichment using polyA tail capture. cDNA libraries were prepared using the NEBNext Ultra II RNA Library Prep Kit from Illumina and sequenced on the Illumina NovaSeq 6000 for 150 bp, paired-end sequencing.

### 2.3. *Montastraea cavernosa* transcriptome assembly and annotation

All bioinformatic analyses were carried out on the Frontera system of the Texas Advanced Computing Center [58]. Raw reads from Novogene were adapter-trimmed and quality-filtered in one step using FastP v. 0.20.1 [59] using the -c flag for base correction and the -x flag for polyA tail trimming. Then, six samples (two from each health state selected at random) were used to generate a de novo metatranscriptome using Trinity v. 2.14.0 [60]. Non-coral transcripts were filtered out of this metatranscriptome using the *in silico* filtration method outlined previously [61]. First, the longest transcript isoform was obtained using the get_longest_isoform_seq_pr_trinity_gene.pl script within the Trinity v. 2.14.0 package [60]. This assembly was then Blasted against a Master Coral database [62] comprising both genome-derived predicted gene models and transcriptomes spanning a wide diversity of coral families using BlastX v. 2.2.27 [63]. Transcripts with less than 95% identity of this Master Coral database and shorter than 150 bp in length were filtered out of the assembly. TransDecoder v. 5.5.0 [64] was used to generate a predicted protein-coding sequence from the longest open reading frame from each transcript, resulting in a predicted proteome for *M. cavernosa*. Sequences with high similarity were then collapsed using cd-hit v. 4.8.1 [65] under default parameters. The resulting sequences were then extracted from the initial assembly to generate the *M. cavernosa* reference transcriptome (table 1). Finally, the transcriptome was annotated with reviewed UniprotKB/Swiss-Prot Entry IDs using BlastX v. 2.2.27 [63].

**Table 1.** Reference transcriptome assembly metrics.

type	no. contigs	complete and single copy (%)	complete and duplicated (%)	fragmented (%)	missing (%)	N50
*M. cavernosa* assembly metrics based on metazoan reference						
de novo	73 047	76.0	11.3	3.1	9.6	16 467
*C. goreaui* assembly metrics based on eukaryote reference						
de novo	48 013	52.9	16.1	7.1	23.9	13 469

Assembly completeness was assessed via Benchmarking Universal Single-Copy Orthologs (BUSCO) v. 5.2.2 and the ‘bbstats.sh’ script within the BBMap v. 38.90 package [66,67]. Missing (%) refers to the percentage of expected single-copy orthologs that were not detected in the transcriptome assembly, based on the selected BUSCO reference dataset.

### 2.4. Isolation and quantification of holobiont reads

To separate *M. cavernosa* and Symbiodiniaceae reads, BBSplit v. 38.90 [66] was used with the *M. cavernosa* reference transcriptome generated above and Symbiodiniaceae transcriptomes of similar assembly quality representing the genera *Symbiodinium, Breviolum, Cladocopium* and *Durusdinium* sourced from previous publications as binning references [68–71]. The binning statistics output was used to identify *Cladocopium* as the dominant Symbiodiniaceae genus present in each sample (electronic supplementary material, figure S1 and table S1). A predicted proteome was generated from the *C. goreaui* reference transcriptome used above with TransDecoder v. 5.5.0 [64]. Similar sequences in the proteome were collapsed using cd-hit v. 4.8.1 [65]. The resulting sequences were then extracted from their initial assembly to generate the final *C. goreaui* reference transcriptome. The resulting *C. goreaui* assembly was then annotated with reviewed UniprotKB/Swiss-Prot Entry IDs using BlastX v. 2.2.27 [63]. Finally, *M. cavernosa* and *C. goreaui* reads from all 22 samples were mapped to their respective transcriptome and quantified using Salmon v. 1.5.2 [72] under default parameters for *M. cavernosa* reads and a k-mer value of 23 for *C. goreaui* reads.

### 2.5. Feature selection on holobiont gene expression

All data analysis was performed using R v. 4.2.2 [73]. Gene count matrices were generated for the quantified *M. cavernosa* and *C. goreaui* transcripts using Tximport v. 1.16.1 [74]. Annotated *M. cavernosa* and *C. goreaui* genes with an *e*-value < 1.0 × 10^−6^ were kept for DE analyses. Regularized log (rlog) normalized expression was obtained in each dataset using DESeq2 v. 1.38.3 [75] with the design ~*Site + Disease_state,* ensuring that any gene expression differences attributable to the collection site were accounted for in downstream analyses. Additionally, genes with an average rlog expression < 10 were removed. This threshold minimizes noise from non-informative genes and aligns with standard transcriptomic practices [75]. Prior to feature selection, a principal component analysis was performed to assess gene expression differences between the endemic and epidemic sites, and no obvious separation between sites was identified (electronic supplementary material, figure S5). Therefore, all samples belonging to the same health state (HH, HD or LD) were pooled across sites, resulting in the following sample sizes per health state: HH: *n* = 6, HD: *n =* 8 and LD: *n =* 8. Then, the *sigFeature()* feature selection algorithm within the sigFeature v. 1.16.0 R package [52] was used to produce a ranked list of genes for each health state in *M. cavernosa* and *C. goreaui* (figure 1*d*; electronic supplementary material, tables S2–S7). This algorithm combines SVM-RFE with statistical significance testing (*t-*statistic), ranking genes by their classification accuracy and their DE levels to ensure biological relevance [76].

To evaluate the classification efficacy of features from each tissue health state, we performed external stratified 10-fold cross-validation on the top 400 features from each dataset using the *sigFeature.enfold()* [52] function. This function partitions the dataset (22 samples) into 10 approximately equal-sized folds (two folds with three samples each, and eight folds with two samples each) and ranks the features in each fold based on their contribution to class separation through recursive elimination. For each iteration, nine folds were used for feature selection and training, while the remaining fold was reserved for testing the performance of the selected features. This procedure was repeated 10 times, with each fold serving as the validation set exactly once, ensuring that every sample in the dataset was used for both training and validation.

Following the cross-validation, we applied the *sigFeatureFrequency()* [52] function to rank features based on their frequency of selection across all 10 iterations. This function generated a ranked list of the top 400 features, assigning frequency scores based on how often each feature appeared in the top 400 features of each iteration of cross-validation. We then used these frequency scores to perform a feature sweep analysis using the *sigCVError()* [52] function, which calculates the cross-validation error rates across increasing numbers of the top features (from 1 to 400). The error rates were plotted to visualize both the mean error and the standard deviation across the 10 iterations of cross-validation (figure 1*e*,*f*).

### 2.6. Functional enrichment of top features in *Montastraea cavernosa*

Functional enrichment was performed on both the upregulated and downregulated genes within the top 500 features from each tissue health state in *M. cavernosa* and *C. goreaui*. To find the upregulated features in each tissue health state, log_2_FoldChange values from DESeq2 relative to the two other tissue health states were obtained for the top 500 features. Features with a log_2_FoldChange > 0 relative to the two other tissue health states were classified as ‘upregulated’, while those with a log_2_FoldChange < 0 were classified as ‘downregulated’ (electronic supplementary material, tables S8–S13). For example, to identify upregulated HH features, log_2_FoldChange was obtained for the comparisons between HH and HD corals as well as between HH and LD corals, and those features out of the top 500 HH features that had higher expression in HH corals relative to both HD and LD corals were deemed ‘upregulated’. To perform functional enrichment of those upregulated and downregulated features, first, the predicted proteomes of *M. cavernosa* and *C. goreaui* generated above were uploaded to STRING v. 12.0 [77] using the ‘Add organism’ tool (*M. cavernosa* STRING ID: STRG0A38MOJ, *C. goreaui* STRING ID: STRG0A06ZQW). Then, lists of the upregulated and downregulated features were uploaded to STRING separately. All Gene Ontology, Reactome and Kyoto Encyclopedia of Genes and Genomes (KEGG) terms were saved, and a curated list of the five most relevant and non-redundant upregulated and downregulated terms was plotted.

### 2.7. Expression analysis of potential tissue health state biomarkers

The top 15 features from each tissue health state in both *M. cavernosa* and *C. goreaui* were selected for further analysis due to their high classification potential. rlog values were obtained for each list of the top 15 features in *M. cavernosa* and plotted in relative expression heatmaps. To confirm gene function, protein domain architecture was obtained and analysed for each feature by uploading their protein sequence to InterPro [78] (electronic supplementary material, tables S14–S19). From each tissue health state’s top 15 features, the top feature as well as four other highly relevant features were plotted in boxplots. Additionally, three features from each tissue health state in *C. goreaui* were selected based on their DE significance and functional relevance and were plotted in boxplots.

## 3. Results

### 3.1. Transcriptome assembly and annotation

Sequencing of 22 coral tissue samples resulted in a total of 1.035 billion raw reads with an average of 47 million raw reads per sample. De novo transcriptome assembly of the cleaned and quality-filtered *M. cavernosa* reads resulted in an assembly of 73 047 contigs with an N50 size of 16 467 bp, and filtering of the *C. goreaui* de novo transcriptome published previously [70] resulted in an assembly of 48 013 contigs with an N50 size of 13 469 bp (table 1). Of those, 33 614 *M. cavernosa* and 26 245 *C. goreaui* contigs were annotated with an annotation *e*-value < 1.0 × 10^−6^. The higher percentage of missing genes in the *C. goreaui* reference transcriptome compared with *M. cavernosa* may reflect gaps in existing eukaryotic reference databases.

### 3.2. Isolation and quantification of holobiont reads

A total of 692 million reads were assigned to *M. cavernosa* and 139 million to *C. goreaui*. An average of 31.5 million reads and 6.34 million reads were assigned to *M. cavernosa* and *C. goreaui* per sample, respectively. Mapping of *M. cavernosa* and *C. goreaui* reads to their respective reference transcriptome resulted in average mapping rates of 92.2 and 91.5%, respectively (electronic supplementary material, table S1). Following transcript quantification, a total of 19 039 and 11 289 length-normalized transcripts with an annotation *e*-value < 1.0 × 10^−6^ were expressed in *M. cavernosa* and *C. goreaui*, respectively. Filtering out genes with an average rlog expression < 10 resulted in 17 229 and 2158 genes with significant levels of expression in *M. cavernosa* and *C. goreaui*, respectively.

### 3.3. Feature selection on holobiont gene expression

The 17 229 annotated genes with significant expression in *M. cavernosa* were used to produce the feature-ranked lists from the HH, HD and LD coral gene expression datasets (electronic supplementary material, tables S2–S7). Of the *M. cavernosa* features, 1562 HH, 5286 HD and 5329 LD had significant DE relative to the other two tissue health states (*t*-statistic *p*‐value < 0.05). To avoid overfitting, we limited cross-validation to the top 400 ranked features from each tissue health state. External stratified *k*-fold cross-validation (*k =* 10) showed high classification performance of each tissue health state’s top 400 coral features: within the HH dataset and LD datasets, 0% average misclassification was achieved with the top 390 and 130 features, respectively, and within the HD dataset, 12% average misclassification was achieved with the top 370 features (figure 1*e*). The higher misclassification rate for HD samples may reflect greater biological heterogeneity in visibly healthy tissue on diseased colonies.

The 2158 annotated genes with significant expression in *C. goreaui* were used to produce the feature-ranked lists from the HH, HD and LD symbiont gene expression datasets. Of the *C. goreaui* features, 152 HH, 114 HD and 291 LD had a significant DE relative to the other two tissue health states (*t-*statistic *p*‐value < 0.05). External stratified *k*-fold cross-validation (*k =* 10) showed high classification performance of each tissue health state’s top 400 symbiont features: within the HH and HD datasets, 0% average misclassification was achieved with the top 160 and 210 features respectively, and within the LD dataset, 5% average misclassification was achieved with the top 360 features (figure 1*f*).

### 3.4. Functional enrichment of top features in *Montastraea cavernosa*

**Figure 2.**
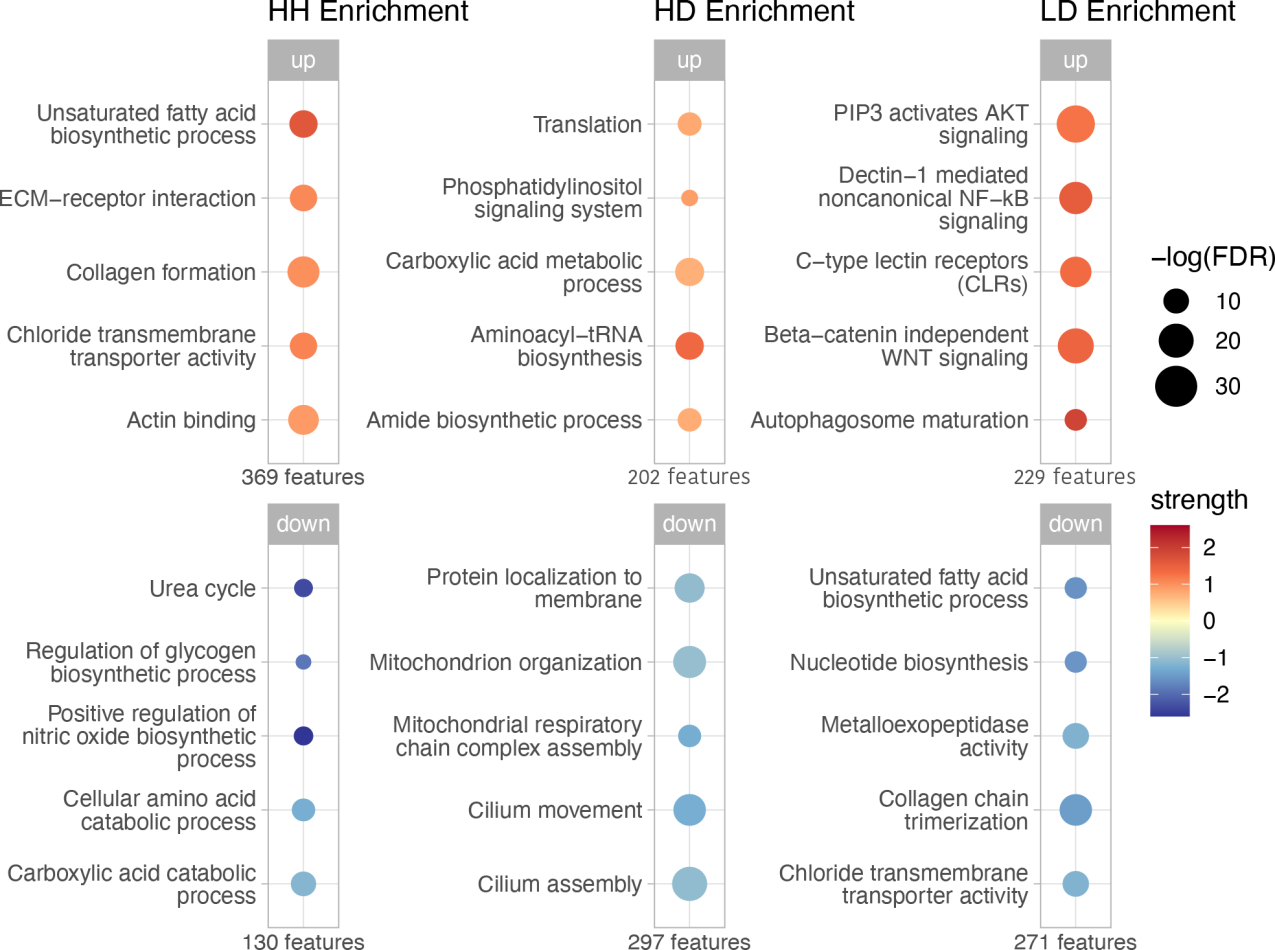
Functional enrichment of upregulated and downregulated genes within the top 500 *M. cavernosa* features from each tissue health state. The number of genes (features) in each list of upregulated and downregulated genes from each tissue health state is shown beneath each bubble plot. Upregulated enrichments are shown on the top panel, and downregulated enrichments are shown on the bottom panel. The size of bubbles represents the log-transformed false discovery rate (FDR) for the enrichment, corrected for multiple testing using the Benjamini–Hochberg procedure. Colour indicates the enrichment strength, calculated as log_10_(observed/expected), where ‘observed’ is the number of genes associated with a function and ‘expected’ is the number expected by chance. Darker colours reflect functions that exceed random expectations. Strength values from the downregulated enrichments were multiplied by −1 for visualization purposes.

In *M. cavernosa*, the top 500 features in each health state revealed clear functional distinctions from the other two disease states. HH tissue was notably enriched for unsaturated fatty acid biosynthesis, collagen formation and actin binding, while HD tissue showed increased translation and amide biosynthesis. In contrast, LD tissue displayed upregulation of innate immune pathways (e.g. C-type lectin (CTL) and NF-κB signalling) and downregulation of collagen chain trimerization and chloride transmembrane transport (figure 2).

**Figure 3.**
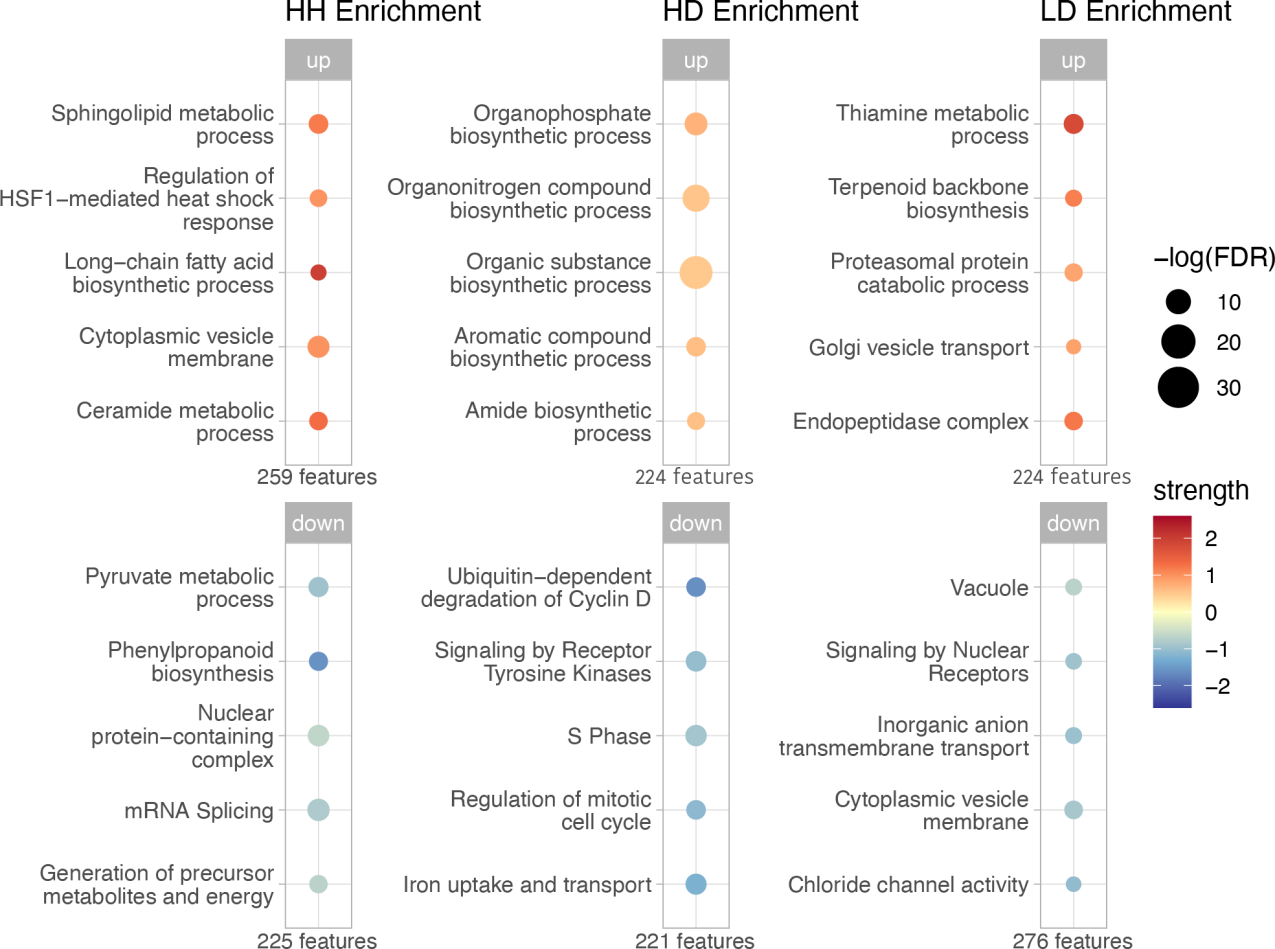
Functional enrichment of upregulated and downregulated genes within the top 500 *C. goreaui* features from each tissue health state. The number of genes (features) in each list of upregulated and downregulated genes from each tissue health state is shown beneath each bubble plot. Upregulated enrichments are shown on the top panel, and downregulated enrichments are shown on the bottom panel. The size of bubbles represents the log-transformed FDR for the enrichment, corrected for multiple testing using the Benjamini–Hochberg procedure. Colour indicates the enrichment strength, calculated as log_10_(observed/expected), where ‘observed’ is the number of genes associated with a function and ‘expected’ is the number expected by chance. Darker colours reflect functions that exceed random expectations. Strength values from the downregulated enrichments were multiplied by −1 for visualization purposes.

The top 500 features in each health state in *C. goreaui* also revealed notable distinctions between health states. *C. goreaui* from HH *M. cavernosa* tissue exhibited enrichment in processes such as long-chain fatty acid biosynthesis, heat shock responses and sphingolipid metabolism, while *C. goreaui* from HD tissue exhibited increased organonitrogen and organophosphate biosynthesis and decreased iron uptake. In contrast, *C. goreaui* from LD tissue was enriched for thiamine metabolism and proteasomal protein catabolism as well as decreased inorganic anion transmembrane transport and chloride channel activity (figure 3).

### 3.5. Expression analysis of potential tissue health state biomarkers

**Figure 4.**
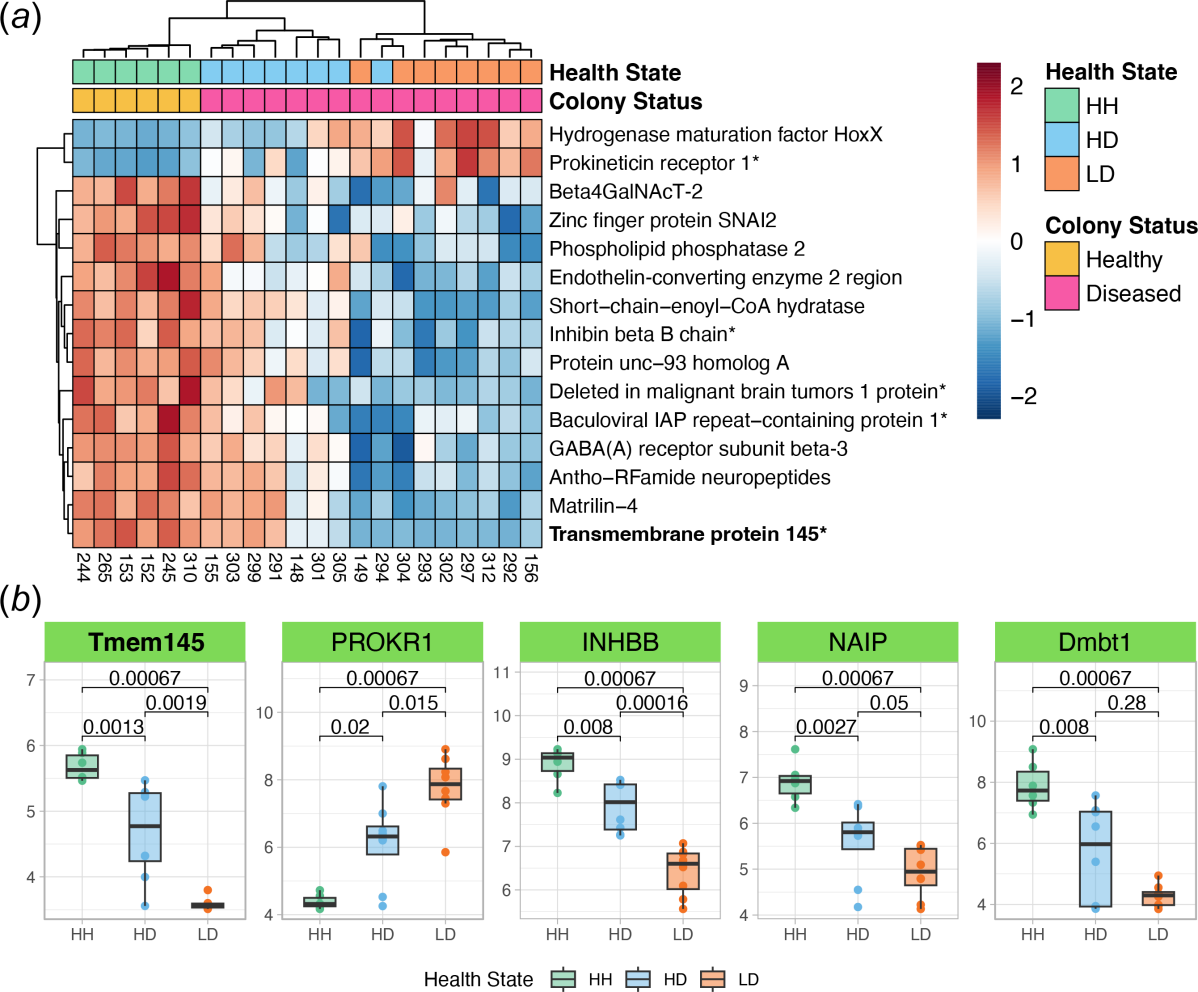
Top features in HH *M. cavernosa.* (*a*) Relative expression heatmap of the top 15 HH features from *M. cavernosa*. Red boxes signify elevated expression relative to the row mean, and blue boxes signify lowered expression relative to the row mean. The top feature is shown in bold, and genes plotted in (*b*) end in an asterisk. (*b*) Boxplots showing the rlog-transformed expression of five selected features from (*a*), organized by tissue health state. *p-*values represent two-sample Wilcoxon test results. The colour of boxplots corresponds to tissue health state. Tmem145: TGFβ signalling; PROKR1: G protein-coupled receptor signalling; INHBB: TGFβ signalling; NAIP: apoptosis regulation; Dmbt1: microbial homeostasis. Boxplot elements: centre line, median; box limits, upper and lower quartiles; whiskers, 1.5× interquartile range; points beyond whiskers, outliers.

**Figure 5.**
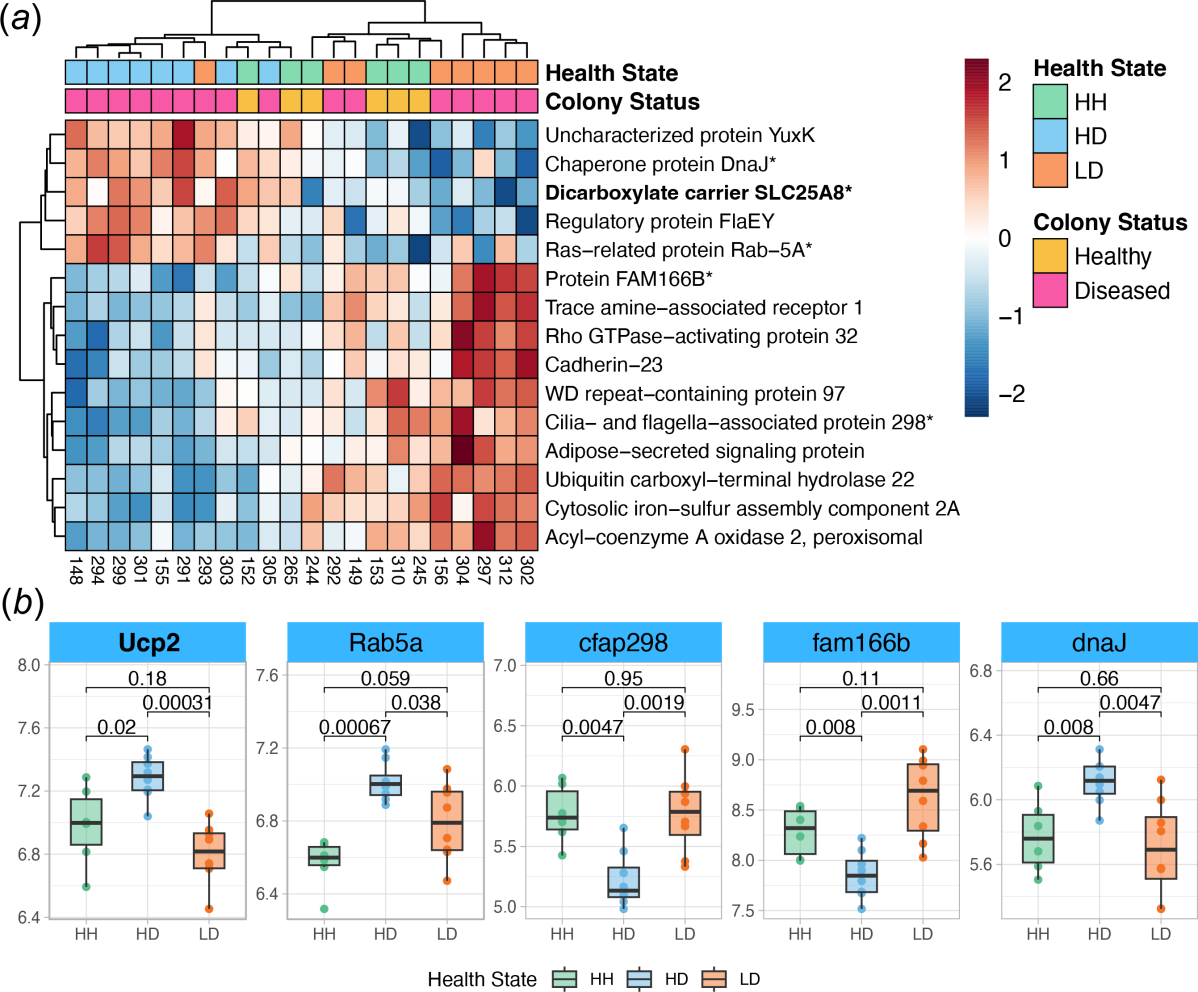
Top features in HD *M. cavernosa.* (*a*) Relative expression heatmap of the top 15 HD features from *M. cavernosa*. Red boxes signify elevated expression relative to the row mean, and blue boxes signify lowered expression relative to the row mean. The top feature is shown in bold, and genes plotted in (*b*) end in an asterisk. (*b*) Boxplots showing the rlog-transformed expression of five selected features from (*a*), organized by tissue health state. *p-*values represent two-sample Wilcoxon test results. The colour of boxplots corresponds to tissue health state. Ucp2: antioxidant; Rab5a: symbiont uptake; cfap298: cilia motility; fam166b: cilia motility; dnaJ: chaperone protein. Boxplot elements: centre line, median; box limits, upper and lower quartiles; whiskers, 1.5× interquartile range; points beyond whiskers, outliers.

**Figure 6.**
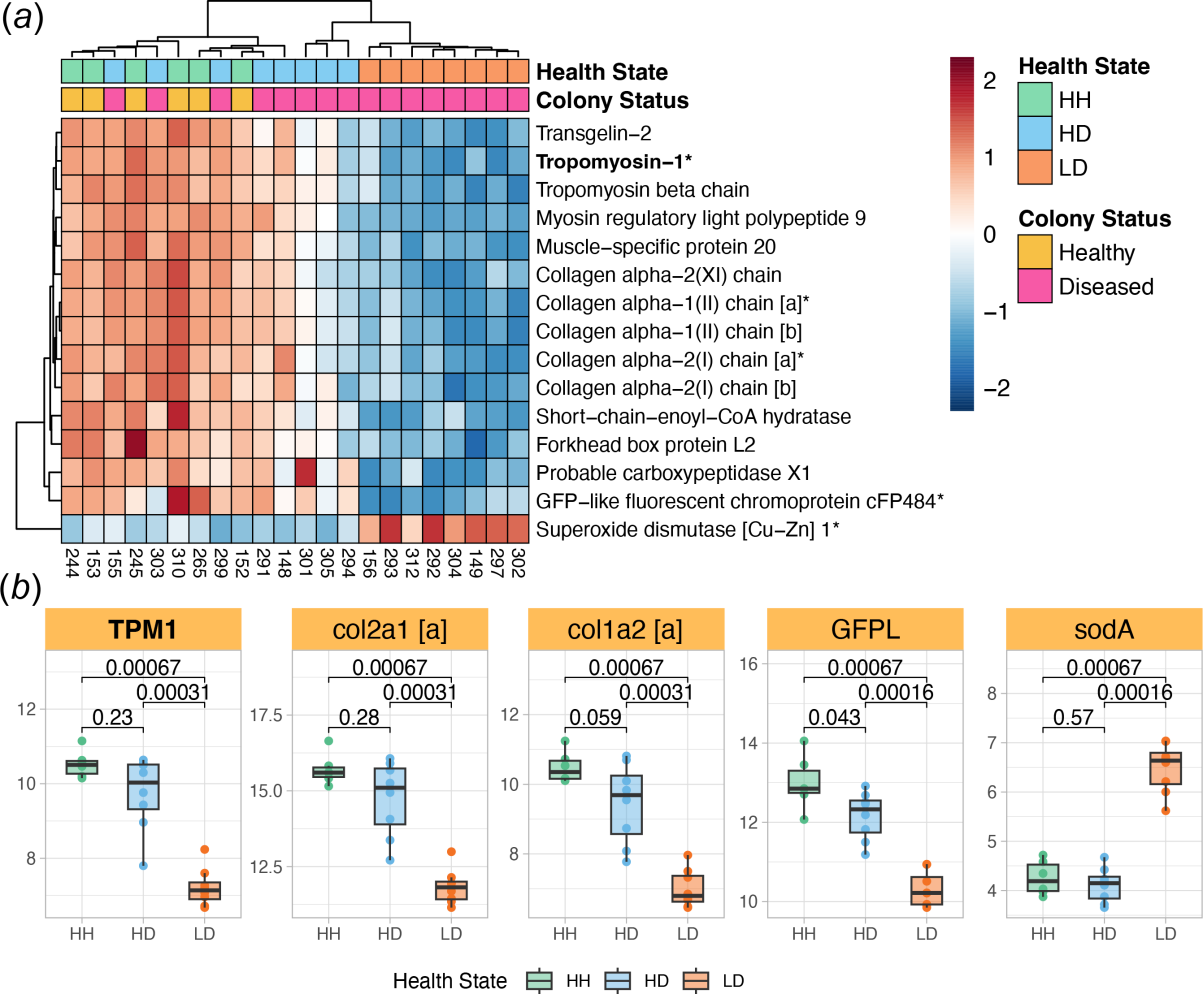
Top features in LD *M. cavernosa.* (*a*) Relative expression heatmap of the top 15 LD features from *M. cavernosa*. Red boxes signify elevated expression relative to the row mean, and blue boxes signify lowered expression relative to the row mean. The top feature is shown in bold, and genes plotted in (*b*) end in an asterisk. (*b*) Boxplots showing the rlog-transformed expression of five selected features from (*a*), organized by tissue health state. *p-*values represent two-sample Wilcoxon test results. The colour of boxplots corresponds to tissue health state. TPM1: cytoskeleton stabilization; col2a1 [a]: ECM structural component; col1a2 [a]: ECM structural component; GFPL: photoprotection; sodA: antioxidant. Boxplot elements: centre line, median; box limits, upper and lower quartiles; whiskers, 1.5× interquartile range; points beyond whiskers, outliers.

We identified the top 15 features in both *M. cavernosa* and *C. goreaui* for each health state based on SVM-RFE rankings, which prioritize genes by their classification power and magnitude of DE (electronic supplementary material, tables S2–S4). In *M. cavernosa,* HH tissue exhibited elevated expression of transmembrane protein 145 (*Tmem145*), an integral membrane component associated with transforming growth factor beta (TGFβ) signalling [78] and other genes implicated in immune regulation and tissue homeostasis, such as prokineticin receptor 1 (*PROKR1*), inhibin beta B chain (*INHBB*), baculoviral IAP repeat-containing protein 1 (*NAIP*) and deleted in malignant brain tumours 1 protein (*Dmbt1*) (figure 4 and electronic supplementary material, table S14). In HD tissue, key upregulated genes included dicarboxylate carrier SLC25A8 (*Ucp2*), a mitochondrial uncoupling protein that may modulate reactive oxygen species (ROS) production [78] and Ras-related protein Rab-5A (*Rab5a*), which is essential for endocytic vesicle trafficking and symbiont uptake [79,80] (figure 5 and electronic supplementary material, table S15). By contrast, LD tissue exhibited marked downregulation of cytoskeletal and ECM-related genes such as tropomyosin-1 (*TPM1*), multiple collagen isoforms, as well as upregulation of superoxide dismutase [Cu-Zn] 1 (*sodA*) (figure 6 and electronic supplementary material, table S16).

**Figure 7.**
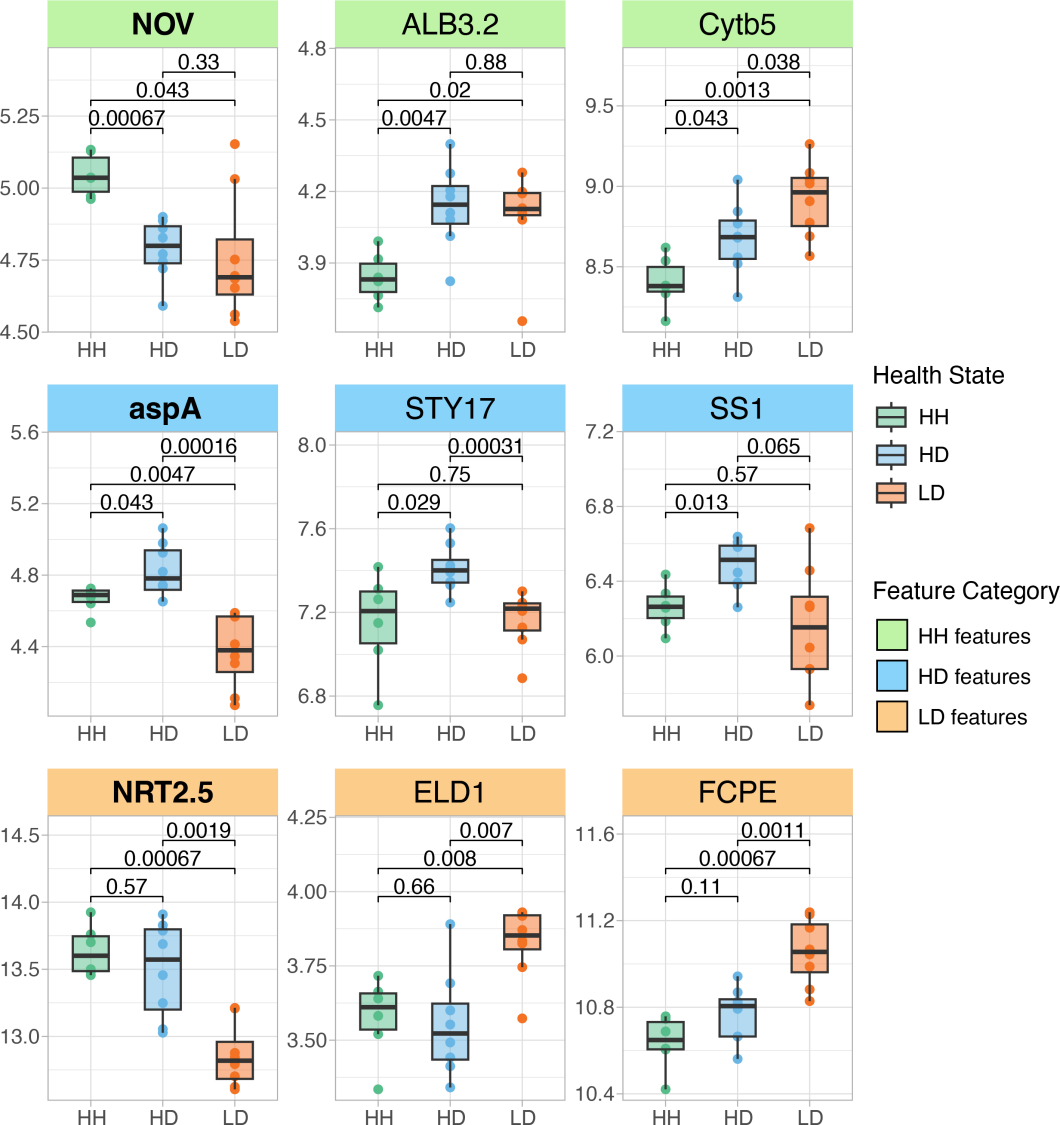
Selected top features in *C. goreaui*. Boxplots show the rlog-transformed expression of three selected features from each tissue health state. HH features are shown in the top panel, HD features in the middle panel and LD features in the bottom panel. The top feature from each tissue health state is shown in the first column in bold. *P-*values represent the two-sample Wilcoxon test results. The colour of boxplots corresponds to tissue health state. Boxplot elements: centre line, median; box limits, upper and lower quartiles; whiskers, 1.5× interquartile range; points beyond whiskers, outliers. Gene functions are listed in electronic supplementary material, table S20.

Of the top 15 HH features in *C. goreaui*, six (40%) were upregulated and nine (60%) were downregulated relative to both HD and LD tissue (electronic supplementary material table S5, figure S3). The top feature in HH *C. goreaui* symbionts was the upregulated protein NO VEIN, which contains a histidine kinase/heat shock protein 90 (HSP90)-like ATPase superfamily domain found in several ATP-binding proteins (electronic supplementary material, table S17) [78]. Other notable genes within the top 15 HH features include inner membrane ALBINO3-like protein 2, chloroplastic (ALB3.2) and cytochrome b5 (*Cyt-b5*) (figure 7). Of the top 15 HD features in *C. goreaui*, seven (46.7%) were upregulated and eight (53.3%) were downregulated relative to both HH and LD tissue (electronic supplementary material table S6, figure S4). The top feature in HD *C. goreaui* symbionts was the upregulated aspartate ammonia-lyase (*aspA*), which converts L-aspartate to fumarate and ammonia during the tricarboxylic acid cycle (electronic supplementary material table S18) [78]. Other notable genes within the top 15 HD features include serine/threonine protein kinase STY17 (*STY17*) and soluble starch synthase 1, chloroplastic/amyloplastic (*SS1*) (figure 7). Of the top 15 LD features in *C. goreaui*, 13 (86.7%) were upregulated and two (13.3%) were downregulated relative to both HH and HD tissue (electronic supplementary material table S7, figure S5). The top feature in LD *C. goreaui* symbionts was the downregulated high-affinity nitrate transporter 2.5 (NRT2.5), which is involved in the transmembrane transport of nitrogen (electronic supplementary material, table S19) [78]. Other notable genes within the top 15 LD features include fucoxanthin-chlorophyll a-c binding protein E, chloroplastic (*FCPE*) and glycosyltransferase-like KOBITO 1 (*ELD1*) (figure 7).

## 4. Discussion

**Figure 8.**
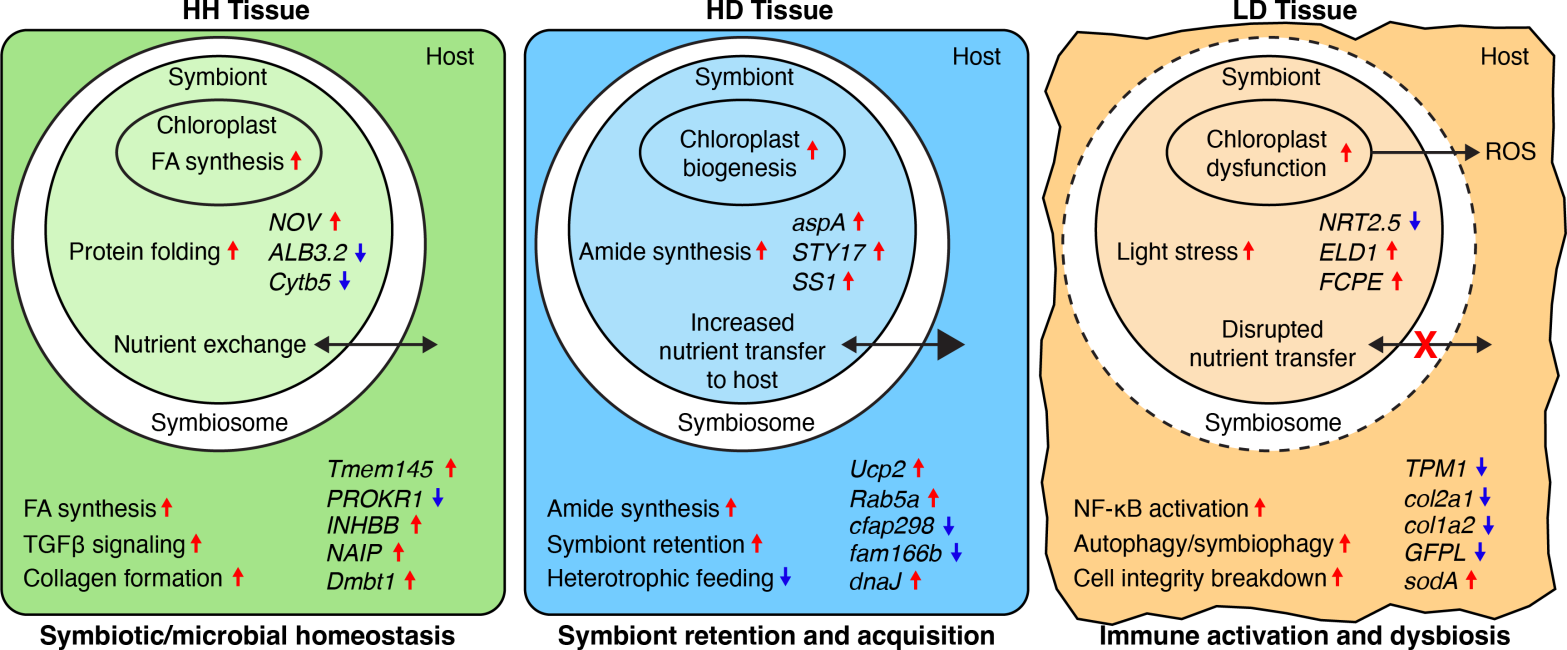
Characterization of SCTLD tissue health states in *M. cavernosa* and *C. goreaui*. Processes are shown in regular font, and selected features are shown in italic font. Processes are inferred from both functional enrichment of the top 500 features and analysis of selected features (genes listed). Upregulated processes/features are denoted with a red arrow, and downregulated processes/features are denoted with a blue arrow. (FA, fatty acid; ROS, reactive oxygen species).

Supervised ML is a powerful tool that uses known characteristics to detect meaningful patterns within large, unstructured and complex datasets [81]. To our knowledge, this is the first study to apply supervised ML to coral gene expression data to characterize a disease. Traditional DE analyses identify genes with significant expression differences between conditions, but they do not inherently account for multivariate relationships between genes or rank features based on their ability to discriminate between disease states [75,82,83]. In contrast, our approach integrates DE analysis with SVM-RFE, a feature selection algorithm that prioritizes the most informative genes, providing a refined set of biomarkers with high discriminatory power [52]. Here, we used this supervised ML feature selection algorithm to characterize distinct SCTLD health states in the reef-building coral, *M. cavernosa*, and its dominant algal endosymbiont, *C. goreaui,* in the USVI (figure 8). Our analysis identified key molecular signatures distinguishing HH, HD and LD. The high classification accuracy of each dataset’s top features (figure 1) demonstrates the effectiveness of ML-driven feature selection in reducing noise and focusing on biologically relevant disease markers. These findings provide novel insights into the processes involved in SCTLD pathogenesis in the USVI.

### 4.1. Novel application of feature selection for characterizing coral health states

Coral research has inherent challenges, one of the most limiting being the low availability of samples from vulnerable or threatened populations, and the low availability of these samples exhibiting active pathological signs of a specific disease or health state. While it is preferable to use large sample sizes to draw robust conclusions in gene expression studies, it is often not possible. Keeping this in mind, the methodology presented in this study leverages a supervised ML-based feature selection algorithm and validation procedure that is designed to identify important gene expression signatures even from limited sample sizes.

We selected sigFeature as our feature selection algorithm because of its ability to address the challenges posed by small-sample gene expression studies [52]. By combining SVM-RFE, which excels at handling high-dimensional data [84], with the DE *t*-statistic to rank genes, sigFeature ensures that selected features are both discriminative and biologically meaningful. This methodology aligns with emerging approaches that aim to combine ML techniques with biological knowledge to enhance biomarker discovery [76]. Additionally, the ability of sigFeature to handle unbalanced class distributions and high-dimensional data makes it well-suited for coral gene expression studies. Furthermore, the feature selection process was rigorously evaluated using external stratified 10-fold cross-validation, a standard technique for assessing classifier model performance [85,86]. This cross-validation method operates independently of the feature selection process and provides unbiased performance metrics across all folds, offering a reliable estimate of feature classification importance. In this study, cross-validation results were consistent with those reported in larger dataset studies [52] and exhibited stable performance across all tissue health states.

### 4.2. Apparently healthy tissue on an apparently healthy colony tissue exhibited signatures of fatty acid biosynthesis and markers of symbiotic stability

Our results showed that metabolism within apparently healthy tissue on apparently healthy colonies was broadly characterized by high levels of fatty acid biosynthesis in both *M. cavernosa* and *C. goreaui*. Fatty acid biosynthesis is critically important for various biological processes, such as maintaining membrane structure and function, energy storage and metabolism and cell signalling [87]. Symbiodiniaceae have been shown to supply their coral hosts with myriad saturated and polyunsaturated fatty acids [88], and previous work has found that Symbiodiniaceae-derived lipid content is higher in apparently healthy *M. cavernosa* corals as opposed to SCTLD-affected corals [47]. The enrichment of fatty acid biosynthesis in both the coral host and its algal symbiont in apparently healthy tissue further emphasizes the importance of this metabolic pathway in maintaining overall health and symbiotic homeostasis.

Within the top *M. cavernosa* HH features, we found high expression of genes involved in TGFβ signalling (*Tmem145* and *INHBB*). The TGFβ signalling pathway is conserved throughout metazoan evolution and functions in a variety of processes such as development, cellular homeostasis and immune regulation [89]. In symbiotic cnidarians, TGFβ signalling is known to suppress host immunity to establish and maintain symbiosis with Symbiodiniaceae [90–92]. Notably, previous transcriptomic studies of SCTLD-affected corals from both Florida and the USVI have consistently reported higher expression of TGFβ signalling genes in apparently healthy individuals compared with their diseased counterparts [48–50], suggesting a conserved host response that may contribute to resistance by regulating inflammation. High constitutive levels of TGFβ signalling, therefore, may constitute an important mechanism of SCTLD resistance through the maintenance of symbiosis and prevention of a detrimental inflammatory response. Our findings highlight the potential utility of monitoring TGFβ signalling as a biomarker for coral health and resilience during SCTLD outbreaks.

Indeed, another upregulated gene within the top *M. cavernosa* HH features included *Dmbt1*, known in humans to be involved in maintaining mucosal homeostasis by preventing bacterial invasion and suppressing inflammation [93]. Previous transcriptomic studies in marine invertebrates have shown differential regulation of *Dmbt1* expression in response to both bacterial challenge and symbiont acquisition [94–97], supporting the hypothesis that this gene contributes to maintaining healthy associations with commensal microbes [97]. Notably, the present study (as well as our prior SCTLD transmission experiment [48], both involving USVI corals) consistently found higher *Dmbt1* expression in healthy corals relative to diseased corals, whereas Traylor-Knowles *et al.* [49] observed the opposite pattern in SCTLD-affected corals from Florida. This discrepancy may be attributable to region-specific variations in SCTLD pathology. Work *et al.* [98] recently highlighted distinct histopathological differences between Florida and USVI corals affected by SCTLD: USVI lesions are characterized predominantly by mucus cell hypertrophy with minimal mesogleal involvement and few viral-like particles, whereas Florida lesions exhibit more extensive tissue degradation [39]. These different disease presentations may underlie the divergent *Dmbt1* expression patterns observed across studies, suggesting that *Dmbt1* could serve as a region-specific indicator of SCTLD pathophysiology. Further investigations are warranted to determine both the function of *Dmbt1* in corals as well as its specific role in localized disease responses.

The top feature in HH *C. goreaui* annotated to protein NO VEIN (*NOV*), a plant-specific nuclear factor required for cell-fate decisions in *A. thaliana* [99]. Interpro protein domain analysis found a histidine kinase/HSP90-like ATPase superfamily domain within this gene’s protein-coding sequence. This conserved domain is found in proteins involved in both cellular signalling and protein folding through the histidine kinase domain and the HSP90-like ATPase domain, respectively [78]. Histidine kinases are responsible for triggering signal transduction in response to environmental stimuli, such as nutrient availability, pH, osmolarity and light, while HSP90-like ATPases are molecular chaperones that play an essential role in protein folding and stabilization [78]. Although *NOV* is plant-specific, the presence of these conserved domains suggests that its role in *C. goreaui* may parallel stress-response mechanisms in plants. High expression of this gene in *C. goreaui* from apparently healthy coral tissue may reflect enhanced environmental sensitivity and protection against protein misfolding.

### 4.3. Apparently healthy tissue on a stony coral tissue loss disease-affected colony tissue showed signatures of symbiont acquisition and retention in response to stony coral tissue loss disease

Although visibly healthy tissue on SCTLD-affected colonies has been shown to exhibit cellular morphological changes associated with Symbiodiniaceae pathology, such as chloroplast deformity and cavity formation [39], the molecular mechanisms underlying these early stage responses remain poorly understood. Here, we leveraged a supervised ML approach to identify genes that were strongly upregulated or downregulated specifically within visibly healthy tissue on diseased colonies (HD). Using a supervised feature selection algorithm allowed us to pinpoint specific molecular signatures that distinguish HD tissue as a biologically distinct state rather than just an intermediate phase between healthy and diseased tissue.

One of the most notable findings in HD *M. cavernosa* tissue was the increased expression of *Rab5a*, a gene encoding a small GTPase critical for early endosome formation and phagocytosis [100]. In cnidarians, Rab proteins play a fundamental role in symbiosis by mediating endosomal trafficking and symbiosome stabilization within host cells [80]. Specifically in the sea anemone *Aiptasia pulchella* (*Exaiptasia diaphana*)*, Rab5a* has been shown to localize to symbiosomes only when they contain viable, newly acquired Symbiodiniaceae, but not when symbionts are damaged or degraded [79]. The upregulation of *Rab5a* in HD tissue suggests that healthy tissue on diseased colonies may be actively increasing symbiont uptake and retention, potentially as a compensatory response to disease exposure. However, this response may not necessarily be beneficial, as many intracellular pathogens, including some bacterial and protozoan parasites, exploit *Rab5a*-mediated trafficking to hijack host endocytic pathways, avoiding degradation while persisting inside host cells [101–103]. If SCTLD involves a microbial pathogen that manipulates host cellular machinery, the observed *Rab5a* upregulation in HD tissue could indicate early stage pathogen interference with host immune or symbiont regulation pathways. Alternatively, if SCTLD is driven in part by compromised or dysfunctional Symbiodiniaceae, increased *Rab5a* expression may facilitate retention of impaired symbionts, leading to host stress and accelerating disease progression. Regardless of the underlying cause, these findings support evidence that SCTLD-associated molecular disruptions occur prior to visible tissue loss [36,39]. Future studies should explore whether *Rab5a* upregulation in HD tissue serves as a predictive biomarker for early SCTLD progression and whether interventions targeting host–symbiont interactions could mitigate disease impact.

Functional enrichment of HD tissue features revealed a shift in metabolic activity between the coral host and its symbionts during the early onset of SCTLD. In contrast to the high levels of fatty acid metabolism seen in the HH tissue, HD tissue exhibited significant upregulation of amide biosynthesis in both *M. cavernosa* and *C. goreaui*. Given that amides are essential components of proteins, enzymes and signalling molecules, their increased biosynthesis probably reflects a heightened cellular demand for amino acids, potentially to support stress-response protein synthesis or cellular repair mechanisms [104]. In addition, HD *M. cavernosa* exhibited downregulation of genes involved in cilia movement and assembly (*cfap298* and *fam166b*). In corals, cilia are directly involved in heterotrophic feeding and nutrient acquisition [105], and the downregulation of *cfap298* and *fam166b* may indicate a reduction in host nutrient acquisition from the surrounding seawater. This may represent a physiological trade-off, where decreased feeding efficiency is compensated by an increased dependence on symbiont-derived energy production. Concurrently, HD *C. goreaui* exhibited upregulation of *STY17*, a serine/threonine protein kinase essential for chloroplast protein transport and photosynthetic function. In *A. thaliana*, loss of *STY17* resulted in delayed chlorophyll assimilation and reduced photosynthetic capacity [106], implying that its upregulation in HD tissue may reflect a compensatory increase in photosynthetic activity by *C. goreaui*. Taken together, these findings suggest that SCTLD-affected colonies experience early metabolic shifts in visually healthy tissue, potentially marked by a reduced reliance on heterotrophic feeding and an increased dependence on symbiont-derived energy production.

### 4.4. Lesion tissue on a stony coral tissue loss disease-affected colony tissue displayed increased immune activity, extracellular matrix degradation and host–endosymbiont dysbiosis

Functional enrichment of LD *M. cavernosa* features revealed upregulation of genes involved in innate immunity, notably CTL receptor binding and NF-κB signalling pathways. CTLs, identified in many cnidarian species [107], are immune receptors that detect threats by recognizing signals from microbes (microbe-associated molecular patterns) or damaged host cells (damage-associated molecular patterns), activating immune responses through NF-κB signalling and the lectin complement cascade [108]. In symbiotic cnidarians, CTLs also recognize specific glycan patterns on the surface of Symbiodiniaceae cells during symbiosis establishment [109]. Therefore, increased CTL receptor activity in SCTLD lesion tissue may reflect heightened detection of pathogenic microbes, cellular damage from inflammation or disruptions to the coral–symbiont relationship. Additionally, NF-κB signalling mediates a wide array of host responses, including phagocytosis, inflammation and antimicrobial mechanisms, and often promotes cell survival and inhibits apoptosis [110]. Similar to our findings, corals exposed to SCTLD *ex situ* showed elevated expression of NF-κB pathway genes, but this response was suppressed following amoxicillin treatment that slowed or halted lesion progression [50], suggesting bacterial involvement in NF-κB activation. However, Symbiodiniaceae are also known to suppress host NF-κB signalling to maintain symbiosis [111–114], but this suppression can become dysregulated under pathogenic stress. For example, in the jellyfish *Cassiopea xamachana*, symbiotic animals showed lower baseline NF-κB expression than their aposymbiotic counterparts, but following bacterial exposure, only the symbiotic animals strongly upregulated NF-κB, probably triggering a more damaging immune response that led to lower survival [115]. Regardless of the exact stimulus causing NF-κB upregulation in SCTLD lesions, the ability to regulate this immune response and prevent runaway inflammation may be crucial for halting disease progression, positioning the NF-κB pathway as a promising target for SCTLD therapeutic interventions.

Additionally, LD *M. cavernosa* exhibited enrichment of ‘autophagosome maturation’ signatures, suggesting elevated levels of autophagy in lesion tissue. Autophagy, an ancient innate immune defence mechanism in animal cells, is the process by which intracellular pathogens and damaged organelles are delivered to lysosomes for degradation and recycling [116]. In cnidarians, autophagy of Symbiodiniaceae (symbiophagy) occurs when the host-derived symbiosome is transformed from an arrested state of phagocytosis into a digestive vesicle [117]. Notably, *Rab7*, an established marker of symbiophagy, was upregulated in multiple species in response to SCTLD infection, implicating symbiophagy in the pathology of SCTLD [48]. While *Rab7* was not among the top LD *M. cavernosa* features, the enrichment of autophagy-related pathways provides further evidence that lesion tissue may be experiencing either (i) activation of an autophagic immune response against bacterial or viral pathogens, (ii) activation of symbiophagy in response to dead or dysfunctional Symbiodiniaceae, or (iii) both processes occurring concomitantly. The significance of autophagosome maturation in SCTLD-affected corals further highlights the intricate relationship between innate immunity and symbiosis maintenance during the pathogenesis of SCTLD and represents a promising target for further research in disease prevention and intervention efforts.

We also found that SCTLD lesion tissue in *M. cavernosa* was predominantly characterized by a dramatic downregulation of genes involved in maintaining ECM integrity and actin cytoskeletal structure, suggesting significant structural disruption within affected tissues. The most downregulated gene was *Tpm1*, a gene implicated in stabilizing cytoskeletal actin filaments in non-muscle cells. Interestingly, Beavers *et al.* [48] reported significant downregulation of a related gene, tropomyosin 4 (*Tmp4*), across five coral species following SCTLD exposure, indicating a conserved mechanism of cytoskeletal disruption associated with SCTLD lesion formation. Additionally, similar cytoskeletal disruptions have been documented during thermal stress and bleaching events in the closely related coral *Orbicella faveolata* [96]. Together, these observations underscore cytoskeletal destabilization as a potential common pathway contributing to lesion pathology, probably exacerbated by oxidative stress and impaired nutrient exchange resulting from symbiotic breakdown.

Evidence of ECM degradation was observed in the downregulation of five collagen genes (*col11a1,* two *col2a1* homologs and two *col1a2* homologs). These results contrast with Traylor-Knowles *et al.* [49], who observed both upregulation and downregulation of collagen genes in Florida corals experimentally exposed to SCTLD, leading to their hypothesis that corals initially activate wound-healing mechanisms in response to SCTLD-induced tissue degradation [49]. In contrast, our USVI samples collected *in situ* exhibited drastic downregulation of ECM components, indicating a compromised ability of advanced-stage SCTLD-affected corals to maintain ECM integrity. The observed differences in ECM gene expression between the studies probably reflect multiple influencing factors, including disease progression stage (early versus advanced), environmental conditions, geographic location and experimental versus natural disease exposures. Recognizing and understanding how these factors collectively shape coral responses across different temporal and spatial scales is essential for informing targeted interventions and conservation strategies to mitigate SCTLD impacts effectively.

Examining host and endosymbiont features together, we found evidence of photosystem dysfunction, ROS production and dysbiosis in the SCTLD lesion tissue. Notably, the upregulation of *EDL1*, a glycosyltransferase-like protein that acts as a negative regulator of photomorphogenesis, was observed in *C. goreaui* within the lesion tissue. This finding represents a novel insight into the response of *Cladocopium* symbionts to SCTLD, indicating a potential redirection of cellular resources away from growth-related processes. In addition, *FCPE*, a component of the light-harvesting complex embedded in the thylakoid membrane, was also upregulated in *C. goreaui* from the lesion tissue, possibly representing a stress response aimed at enhancing photosynthetic efficiency. However, in *M. cavernosa*, we found downregulation of *GFPL*, a green fluorescent protein-like pigment that is implicated in photoprotection of Symbiodiniaceae [118], as well as a drastic upregulation of *sodA,* a superoxide dismutase that mitigates oxidative damage in corals [119,120]. These patterns suggest that host photoprotective mechanisms were compromised in lesion tissue, exposing symbionts to increased light stress, generating ROS and triggering antioxidant defences. These gene expression signatures resemble known bleaching-associated stress responses [121] and represent further evidence of host–endosymbiont dysbiosis within SCTLD lesion tissue.

## 5. Conclusion

Our gene expression profiling of various tissue health states in *M. cavernosa* and its dominant algal endosymbiont, *C. goreaui*, supports evidence that SCTLD causes dysbiosis between the coral host and its Symbiodiniaceae [32–34,48,50,122]. While this study does not confirm the etiologic agent of SCTLD, it does highlight the unique shifts in host–endosymbiont functioning both during the onset of colony infection and during lesion progression. HD appears to be mounting a response to colony infection by promoting Symbiodiniaceae uptake and retention, possibly to increase autotrophic nutrient acquisition to prepare an immune response. If SCTLD is caused by a pathogen of Symbiodiniaceae, this strategy would be detrimental to the coral host as it could inadvertently increase the acquisition of affected endosymbionts. Lesion tissue, alternatively, showed strong signals of runaway inflammation, loss of cellular integrity and an inability to maintain symbiotic homeostasis. Some of the gene expression signatures in the lesion tissue resemble bleaching responses, such as disruption of actin cytoskeleton structure and oxidative stress in the coral and photosystem dysregulation in the algal endosymbiont. The compounded stress of microbial dysbiosis, combined with a heightened inflammatory response and a breakdown in host–endosymbiont physiology may collectively drive the rapid tissue loss observed in SCTLD progression.

While our research offers important insights into SCTLD disease mechanisms, several factors should be considered when interpreting these findings. Our study focused on a single coral species (*M. cavernosa*) and its dominant algal symbiont (*C. goreaui*) from the USVI, meaning that our results do not fully capture the diversity of SCTLD manifestations across different coral hosts, symbiont types and geographic regions. Additionally, gene expression in corals is highly sensitive to environmental conditions, which vary seasonally and spatially. The results presented here reflect the molecular state of *M. cavernosa* at the specific time of sampling (February 2020), and it is possible that gene expression profiles would differ under different environmental conditions, such as during periods of heat stress or gametogenesis. Furthermore, our sample size was limited due to the availability of suitable coral colonies. Despite this, our use of SVM-RFE allowed us to derive meaningful insights from the available data, and the consistency of gene expression patterns within each health state supports the robustness of our findings. Future studies should aim to incorporate seasonal sampling across regions, a broader range of coral species and larger sample sizes to further assess the extent to which SCTLD-associated gene expression patterns are conserved across environmental conditions.

Our bioinformatic pipeline utilizing the sigFeature pipeline [52] represents the first application of supervised ML feature selection to coral transcriptomics. This study offers a novel framework for characterizing disease pathogenesis in corals and their algal endosymbionts by isolating the most biologically relevant genes with high classification power. With the increasing availability of high-throughput data, these methods can be integrated into omics analyses to accurately characterize various coral physiological states, such as white plague disease, bleaching and emerging disease outbreaks. With this feature selection framework, we can better understand the health states of endangered coral species to develop effective and long-lasting management efforts.

## Data Availability

All raw sequence data and associated sample metadata are deposited in the NCBI SRA database (BioProject PRJNA1062758) and on BCO-DMO [123]. Publicly available data used in this study include the reference transcriptomes for Symbiodinium CassKB8 (BioProject PRJNA80085), *Breviolum minutum* (BioProject PRJNA274852), *Cladocopium goreaui* (BioProject PRJNA307543) and *Durusdinium trenchii* (BioProject PRJNA508937). The Master Coral database used is available in a public Zenodo repository [62]. Additional sampling information and all code used in this study are available on GitHub at [124], and we used Zenodo to assign a DOI to the repository [125]. Supplementary material is available online [126].
